# Tennessee State Dental Association

**Published:** 1869-01

**Authors:** 


### CORRESPONDENCE.

### ARTICLE III.

### *Tennessee State Dental Association.*

This body began its semi-annual meeting in Nashville on Wednesday, December 16th at 10 A. M., at the office of Dr. W. H. Morgan, Vice President Dr. S. J. Cobb in the chair, and Dr. H. M. Acree, Secretary *pro tem.*

There were delegates from the various sections of this State, and representatives from Mississippi, Alabama and Kentucky; among the latter we notice the names of Dr.

S. P. Cutler, of Holly Springs, Miss., and Dr. W. G. Redman, of Louisville, Kentucky.

The first day, after organization, was occupied principally with the discussion of Dental Chemistry and Histology. On Thursday, at the office of Dr. S. J. Cobb, the subjects of discussion were Alveolar Abscess and Diseased Gums, with a clinic by Dr. W. H. Morgan, and the afternoon session was consumed by a very able and interesting paper from Dr. S. P. Cutler, of Miss., on Physiology. At night there was a microscopic investigation at the office of Dr. L. C. Chisholm, conducted by Dr. Cutler.

It was announced that there would be a clinic held on Friday morning at 9 o'clock, at the office of Dr. W. H. Morgan, and that at 10 A. M., at the office of Dr. L. C. Chisholm, several important practical subjects would be discussed. Regular physicians and dentists were cordially invited to attend.

*Third Day—Morning Session.*—Clinic at the office of Dr. Morgan, conducted by Dr. Redman, of Louisville, Kentucky, and Dr. Jordan, of Huntsville, Alabama. The society was called to order at 10 o'clock, at the office of Dr. Chisholm, when on motion discussions on the subject of Microscopy were closed and the subject of Mechanical Dentistry upon which a very animated discussion arose upon the comparative merits of gold, rubber, continuous gum, in which Drs. Morgan, Redman, Chisholm and others participated.

Dr. Love, of Louisville, introduced to the notice of the society Dr. McCleland's new base "Rose Pearl" or collodion setting forth its merit, and claiming main points of superiority for it.

At this point the regular order of business was suspended and a vote of thanks was tendered to the Nashville Medical Society for the courteous offer of their hall to this society during its session: also a vote of thanks to the Union and American and Banner for courtesies extended.

The order of business was resumed until half past one o'clock, when the Association adjourned to meet on Lookout Mountain, July 20, 1869.

This meeting was one of great interest to the profession in attendance, and was largely contributed to by visiting members from the adjoining States. The utmost harmony and good feeling prevailed throughout.

